# *Caenorhabditis elegans* as a Model Organism to Evaluate the Antioxidant Effects of Phytochemicals

**DOI:** 10.3390/molecules25143194

**Published:** 2020-07-13

**Authors:** Begoña Ayuda-Durán, Susana González-Manzano, Ana M. González-Paramás, Celestino Santos-Buelga

**Affiliations:** 1 Grupo de Investigación en Polifenoles (GIP-USAL), Universidad de Salamanca, E-37007 Salamanca, Spain; Bego_Ayuda@usal.es (B.A.-D.); susanagm@usal.es (S.G.-M.); paramas@usal.es (A.M.G.-P.); 2 Unidad de Excelencia Producción, Agrícola y Medioambiente (AGRIENVIRONMENT), Parque Científico, Universidad de Salamanca, E-37185 Salamanca, Spain

**Keywords:** polyphenols, oxidative stress, reactive oxygen species, antioxidant enzymes, signaling pathways, insulin/IGF-1, q-RT-PCR

## Abstract

The nematode *Caenorhabditis elegans* was introduced as a model organism in biological research by Sydney Brenner in the 1970s. Since then, it has been increasingly used for investigating processes such as ageing, oxidative stress, neurodegeneration, or inflammation, for which there is a high degree of homology between *C. elegans* and human pathways, so that the worm offers promising possibilities to study mechanisms of action and effects of phytochemicals of foods and plants. In this paper, the genes and pathways regulating oxidative stress in *C. elegans* are discussed, as well as the methodological approaches used for their evaluation in the worm. In particular, the following aspects are reviewed: the use of stress assays, determination of chemical and biochemical markers (e.g., ROS, carbonylated proteins, lipid peroxides or altered DNA), influence on gene expression and the employment of mutant worm strains, either carrying loss-of-function mutations or fluorescent reporters, such as the GFP.

## 1. Introduction

**Figure 1:**
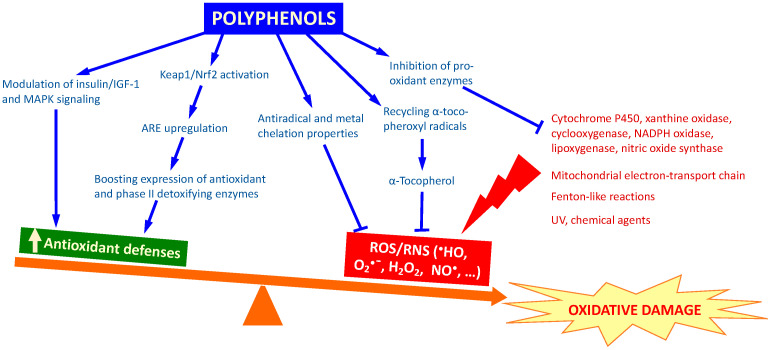
Induction of oxidative damage and its modulation by polyphenols.

During normal metabolism, reactive oxygen and nitrogen species (ROS/RNS), such as hydroxyl (^•^HO), superoxide (O_2_^•^^−^), nitric oxide (NO^•^), nitrogen dioxide (NO_2_^•^), peroxyl (ROO^•^) and lipid peroxyl (LOO^•^) radicals, as well as nonradical species like hydrogen peroxide (H_2_O_2_), hypochlorous acid (HOCl), ozone (O_3_), singlet oxygen (^1^O_2_), and peroxynitrite (ONOO^−^) are constantly generated in organisms. Their main site of production is the mitochondrial electron-transport chain, but reactive species may also originate from the activity of enzymatic systems like cytochrome P450, NAD(P)H oxidases, lipoxygenases, monooxygenases, or nitric oxide synthase (NOS), as well as generated through Fenton-like reactions (Figure 1). Besides, their formation can also be induced by external stressors, such as UV irradiation or chemical agents [1].

At low concentrations, these species play relevant roles as signaling molecules in the regulation of the redox homeostasis in cells and other physiological functions. However, overproduction of ROS results in oxidative stress, leading to damage of cell structures, such as lipids and membranes, proteins, and DNA [1]. As soon as 1956, Harman proposed that the process of ageing was the result of the accumulation of oxidative damage resulting from free radicals [2]. Later on, Sies [3] formulated the “Oxidative Stress Theory”, according to which degenerative diseases could be explained by an imbalance in the dynamic equilibrium between oxidants and antioxidants in the cell (Figure 1).

Several endogenous mechanisms are involved in the regulation of ROS levels in the cell, including antioxidant molecules like glutathione and enzymes like superoxide dismutases (SOD), glutathione peroxidases (GPO) or catalase (CAT) [4]. Besides, dietary antioxidants, such as vitamins E and C, carotenoids or polyphenols have also been indicated to contribute to regulate ROS levels and control oxidative stress [5]. This review will be mainly focused on polyphenols, even though the described methodological approaches are of application to all types of phytochemicals.

## 2. Polyphenols and Oxidative Stress

Phenolic compounds, commonly referred to as “polyphenols”, are one of the most important groups of plant secondary metabolites. They are widespread in higher plants, where they contribute to the mechanisms of natural resistance. They are also widely distributed in the human diet through grains, fruits and vegetables and derived products, such as wine, tea or chocolate, in which they contribute to sensory, technological and health properties. Phenolic acids and flavonoids are the main classes of polyphenols, being the latter most abundant in plants and food. Phenolic acids consist of hydroxybenzoic and hydroxycinnamic acids and their derived esters and glycosides, while flavonoids comprise several groups of compounds, among which flavones, flavonols, flavan-3-ols (i.e., catechins and proanthocyanidins), flavanones, anthocyanins or isoflavones are prominent. Minor polyphenol classes also exist like stilbenes, lignans and phenolic alcohols [6].

Early observations on the beneficial effects of flavonoids were made in the 1930s by the Nobel Prize in Physiology and Medicine winner Albert Szent-Györgyi, who proposed for them a vitamin-like nature (“vitamin P”) [7], a consideration that was maintained for a couple of decades till it was verified that they were not indispensable [8]. The interest in the health properties of polyphenols has recently renewed, as long as epidemiological evidences have accumulated associating their consumption with a lower incidence of mortality from cardiovascular diseases and other degenerative and age-related disorders. Despite they are not nutrients, nowadays dietary polyphenols are considered to contribute to the protective effects of fruit and vegetables in human health [6,9].

Classically the biological effects of polyphenols have been associated to their antioxidant capacity, largely demonstrated in in vitro studies. They behave as effective scavengers of oxidizing species through mechanisms that involve the transfer of an H atom or a single electron to radicals stabilizing them [10]. Besides, they are also able to chelate redox active metal ions, like Fe^2+^ or Cu^2+^, that catalyze Fenton-like reactions, so that removing a causal factor for the production of oxidant species [11]. Furthermore, in vivo, they could also favor indirect antioxidant mechanisms, through inhibition of pro-oxidant enzymes, recycling of α-tocopheroxyl radicals at lipid:water interfaces [12], or activation of natural antioxidant defenses [13].

It seems, nevertheless, unlikely that the in vivo effects of polyphenols can be totally explained by their antioxidant potential. Most polyphenols are little bioavailable and largely biotransformed in the organism, so that their actual concentrations that can be found in plasma and tissues from a dietary consumption are very low, ranging from nanomolar to low micromolar levels [14], far below from those of other physiological antioxidants, like urate, glutathione, α-tocopherol or ascorbate [15]. Furthermore, the compounds able to reach biological targets are usually metabolites chemically distinct from the compounds present in foods [16], and with lower antioxidant activity than the parent compounds [17,18]. Other mechanisms of action should, therefore, contribute to overall polyphenols’ biological effects, among which the modulation of cell oxidative stress through regulation of oxidative stress-sensitive pathways is currently gaining place. Thus, in order to deep into in the mechanisms involved in the molecular effects of polyphenols, the investigation of signaling pathways is crucial.

The evolutionary conserved insulin/insulin like growth factor-1 (IGF-1) signaling pathway controls many important biological processes, such as ageing, metabolism or stress resistance throughout species [19,20]. This pathway has been repeatedly proposed as a key target in the biological effects of different polyphenols [21,22,23,24,25]. Also, the activation of mitogen-activated protein kinases (MAPK) and subsequent inhibition of the mammalian target of rapamycin (mTOR) signaling pathway has been indicated as a mechanism by which polyphenols might influence energy homeostasis and inflammatory processes [13]. Kelch-like ECH-associated protein (Keap1)/Nrf2 (NF-E2 p45-related factor 2) signaling is another pathway that has a crucial role in cell protection, being involved in the regulation of many antioxidant and detoxification genes through the antioxidant response element (ARE) [26,27]. The upregulation of gene expression by induction of the ARE regulatory system is triggered by Nrf2, a transcription factor that can be activated by different phytochemicals [26,27]. Many polyphenols have shown to be able to activate ARE, boosting the expression of antioxidant (e.g., glutathione peroxidase, catalase or superoxide dismutase) and phase II detoxifying enzymes (e.g., NAD(P)H-quinone oxidoreductase, glutathione S-transferase, or UDP-glucuronosyl transferase), which are a major line of defense against oxidative stress [28,29,30]. Similarly, polyphenols may also regulate the oxidative status of the cell by inhibiting oxidative enzymes responsible for superoxide production, such as xanthine oxidase, cyclooxygenase, lipoxygenase, or NADPH oxidase [31]. The inhibition of protein kinase C was, for instance, suggested as a mechanism for the inhibition of NADPH oxidase by flavonoids [32]. A scheme of main mechanisms by which polyphenols may modulate oxidative stress is shown in Figure 1.

## 3. *Caenorhabditis elegans* and Oxidative Stress

Most of the previous mechanisms have been studied using in vitro and cell models, however, it is unclear to what extent they actually contribute to the in vivo effects of flavonoids. In this respect, the nematode *Caenorhabditis elegans* offers promising possibilities for studying the mechanisms subjacent to the biological activity of natural compounds in an in vivo model. Actually, many important molecular pathways in complex organisms can be explored using this worm, as there is a high degree of homology between *C. elegans* and human genes involved in processes like ageing, apoptosis, cell signaling, metabolism, or cell cycle [33,34,35].

In addition to conserve relevant metabolic pathways, this nematode possesses a series of advantages to be used as a model organism. It is non-pathogenic and, as an invertebrate, no ethical boundaries exist to its experimental usage. It has a small size (about 1 mm in length) and a short life cycle (within three days it develops from egg to adult) and lifespan (15–22 days at 25 °C), making it useful for longevity studies [36]. Its reproduction is fast, generating about 300 progenies per self-fertilizing hermaphrodite, and it can be easily propagated in the laboratory on solid or liquid culture media or microtiter plates. It possesses a simple but highly organized organism, with 959 somatic cells in the adult hermaphrodite that are structured in a well characterized system of organs and tissues, including hypodermis, muscle, hypodermis, muscle, reproductive system, a complete digestive tract, and a nervous system consisting of 302 neurons. Furthermore, it is transparent, which allows visualizing processes in the living animal, which can be facilitated with the use of fluorescence probes [37]. *C. elegans* genome is fully sequenced [38] and facilities about genes function are freely available through the WormBase (https://wormbase.org/#012-34-5). Besides, signaling pathways can be manipulated by simple biotechnological methods, and the presence of complete tissue and organ systems also raises the possibility to consider the metabolism of compounds [39].

**Figure 2:**
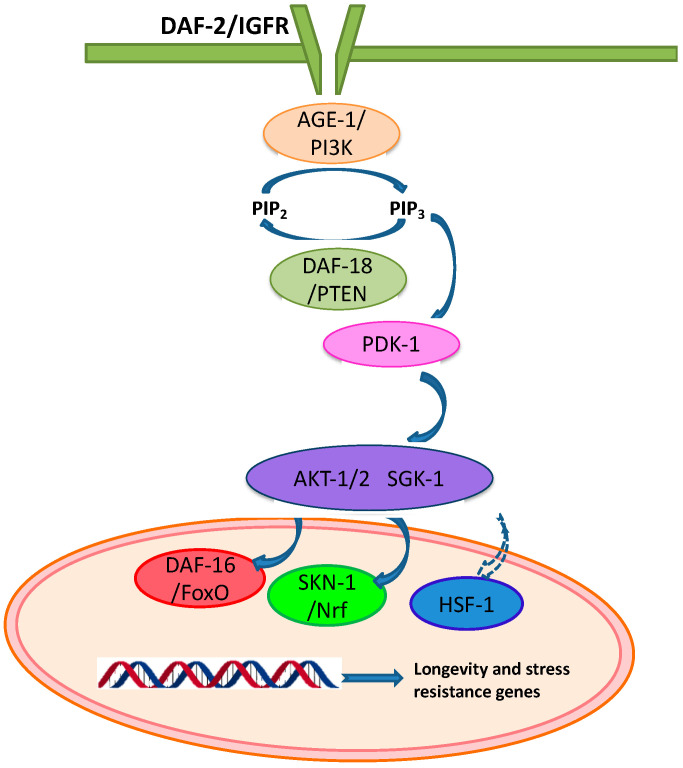
Scheme of the IIS pathway in *Caenorhabditis elegans*.

An important pathway related with the response to oxidative stress in *C. elegans* is the insulin/IGF-1 (IIS) signaling pathway [40]. The IIS pathway (Figure 2) begins with insulin-like peptides (ILPs) binding to DAF-2, the *C. elegans* homologue for the insulin/IGF-1 receptor (IGFR) [41]. DAF-2/IGFR activation triggers a cascade of phosphorylation events through different serine/threonine kinases (AGE-1/PI3K, PDK-1, AKT-1/2 and SGK-1), which results in the phosphorylation of the DAF-16/FoxO, HSF-1, and SKN-1/Nrf transcription factors, preventing their translocation to the nucleus and their transcriptional activity. AGE-1/PI3K signaling is counteracted by the DAF-18/PTEN lipid phosphatase, thus avoiding phosphorylation and cytoplasmic sequestration [42]. Oppositely, the inhibition of the DAF-2 pathway leads to nuclear transport of DAF-16, HSF-1 and SKN-1, changing the expression profile of different genes involved in processes like longevity, stress response, metabolism or protein assembling and refolding, such as catalase (*ctl-1*), superoxide dismutase-3 (*sod-3*), metallothionein (*mtl-1*), bacterial pathogen defense genes (*lys-7, spp-1*), molecular chaperones (e.g., small heat shock proteins like *hsp-16.2*), or glutathione *S*-transferase (*gst-4*) [42,43,44]. HSF-1 binds to DNA specific regions that contain heat shock elements (HSE) resulting in the induction of genes codifying molecular chaperones, like HSP-16 or HSP-70. Members of this protein family are known to be involved in longevity and thermotolerance in *C. elegans*. Thermal stress, for instance, has been shown to increase HSP-16.2 levels [43,45,46,47].

SKN-1, the *C. elegans* ortholog of mammalian Nrf2, belongs to a family of leucine zipper (bZip) proteins in the C-terminal region. This factor, together with other Nrf/CNC proteins, is involved in functions of cell protection, regulating the expression of genes for detoxification (phase II enzymes), antioxidant protection (SOD, GST, GPO, or NQO-1 enzymes) and protein homeostasis (molecular chaperones, protein biosynthesis and protein degradation) [48,49]. When IIS is reduced, the nuclear accumulation and transcriptional activity of SKN-1 is dependent on p38 MAPK signaling [44]. Inactive SKN-1 is constitutively localized in the cytoplasm and it is only translocated to nucleus upon phosphorylation by PMK-1, a mitogen-activated protein kinase homologue of human p38 MAPK, detected in intestinal cells and neurons of *C. elegans*, which is activated when temperature increases above 32 °C. Activation of the transcription factor SKN-1 mediated by the PMK-1 pathway would be, thus, a possible mechanism involved in heat stress response in *C. elegans* [50].

## 4. Methodological Approaches for Antioxidants Evaluation in *C. elegans*

The most direct approach to explore the antioxidant effects in *C. elegans* consists of submitting it to an oxidative challenge, induced either through a chemical or a physical (e.g., heat) stressor, after being treated with a phytochemical. The ability of the compound to reduce oxidative stress can be further evaluated by assessing the survival of the worms after the process and/or through the determination of markers of oxidative stress, comparing with worms submitted to the same conditions but grown in the absence of the phytochemical. Hydrogen peroxide or the redox cycler juglone are the most usually employed chemical stressors, but other compounds like paraquat have also been used. This approach has been applied to evaluate the antioxidant efficiency of polyphenols by many researchers [51,52,53,54,55,56]. Oxidative stress can also be induced by submitting *C. elegans* to a temperature of 32–37 °C [57]. The application of a thermal stress has also been commonly used to assess the antioxidant potential of polyphenols in *C. elegans* [21,22,58,59,60,61,62].

When planning these types of assays, some aspects have to be carefully considered, such as the concentration of the phytochemical in the culture media, time and duration of the treatment, worm age or strength of the oxidative challenge. The assay conditions can greatly influence the results and their interpretation, which might explain the sometimes contradictory results obtained by different authors.

### 4.1. Determination of Markers of Oxidative Damage

#### 4.1.1. Intracellular ROS Levels

The determination of ROS levels is a common approach to evaluate the antioxidant status in *C. elegans*. ROS can be determined after reaction with different dyes, such as 2′,7′-dichlorodihydrofluorescein diacetate (DCFH-DA), MitoTracker^®^ red CM-H(2)XRos or MitoSOX™. In these assays, worms are routinely grown in nematode growth medium (NGM) plates containing the phytochemical and *E. coli* OP50 as a food source; in the day of the assay, they are individually transferred to a well of a multi-well plate, where they are submitted to thermally-induced stress before the reaction with the probe. Fluorescence or chemiluminescence are further measured in a microplate reader. Other techniques that have also been used for ROS measurement after the derivatization reaction are confocal microscopy and electron spin resonance (ESR) [63], fluorescence-activated cell sorting (FACS, a specialized type of flow cytometry) [64], or HPLC with detection by fluorescence, absorbance or mass spectrometry [65].

The choice of the probe is important, as different dyes possess different reaction characteristics. In the case of DFCH-DA, the acetate groups are removed in worm cells and the released DFCH is oxidized by intracellular ROS to yield the fluorescent dye DCF. This reaction is sensitive to H_2_O_2_, ^•^HO and ROO^•^, but not to ^•^NO, HOCl or O_2_^•^^−^ [66]. MitoSOX™ is a dihydroethidium (DHE) molecule that forms fluorescent ethidium upon oxidation by O_2_^•^^−^; although the reaction is considered to be specific for this species, it has also been found that other cell components like cytochrome C are also capable of oxidizing DHE [64]. The Mito Tracker^®^ red probe CM-H2XRos is a reduced form of rosamine used for mitochondrial staining that can be oxidized by ROS, and especially hydrogen peroxide [67]. Similar results in mitochondrial ROS determination were obtained by Kuznetsov et al. [63] with DCF-DA and MitoTracker^®^ red CM-H2XRos using quantitative confocal imaging analysis, although those authors considered MitoTracker^®^ being better for mitochondrial ROS detection.

Amplex red is another probe that can be used to measure H_2_O_2_. The reaction is catalyzed by horseradish peroxidase (HRP) to yield the colored and fluorescent product resorufin. There are, however, confounding side reactions that may alter the results of this assay, such as the light-mediated photochemical oxidation of resorufin in the presence of biological reductants (glutathione, NADH), which artifactually increases the generation of H_2_O_2_ and can also lead to the formation of O_2_^•^^−^, whose reaction with HRP may further affect the stoichiometry of the reaction [68]; this problem that can be overcome by addition of Cu/Zn-SOD to the assay medium [69]. Amplex red does not detect intracellular H_2_O_2_, so that the method is suitable for measuring H_2_O_2_ in extracellular media or in isolated mitochondrial preparations under conditions that limit the secondary radical reactions [68]. This probe was used by Xiong et al. [70] to study the influence of epigallocatechin-3-gallate (EGCG) on ROS production and ageing in *C. elegans*. Table 1 summarizes the characteristics of the different probes for ROS determination.

#### 4.1.2. Glutathione Levels

The redox environment in cells is established by low-molecular mass and protein-bound thiols, being glutathione (γ-glutamylcysteinylglycine) the major and most important intracellular redox buffer [71]. Under normal physiological conditions most of the redox-active glutathione molecules are in the reduced form (GSH) and only a minor fraction is present as glutathione disulfide (GSSG). The GSH/GSSG couple contributes to the maintenance of the reduced intracellular milieu [72] and can be used as an indicator of the redox status in *C. elegans* [73]. In its reduced form, glutathione can directly reduce substrates, but it also acts indirectly through the glutaredoxin system and intervenes in detoxification and repair processes in combination with glutathione *S*-transferase [74].

The most usual way to analyze GSH is based on the use of the Ellman’s reagent (5,5′-dithiobis-(2-nitrobenzoic acid); DTNB), originally proposed for quantification of thiol groups [75] and later optimized to the determination of glutathione in biological samples, including worm homogenates [72,76]. The DTNB reacts with GSH to produce the yellow chromophore 5′-thio-2-nitrobenzoic acid (TNB) that can be measured spectrophotometrically at 412 nm. The method can be applied to the determination of total glutathione by recycling the oxidized form (GSSG) to GSH by glutathione reductase in the presence of NADPH [77,78].

GSH has also been analyzed in *C. elegans* by HPLC with detection after derivatization with *ortho*-phtaldialdehyde (OPA) that yields a stable fluorescent product that can be quantified at 420 nm, as emission wavelength, after excitation at 340 nm [71,72]. Another alternative method proposed for the determination of glutathione in erythrocytes is based on the modification of the chemiluminescence signal resulting from the oxidation of luminol by sodium periodate in basic solution [79], although in our knowledge it has not been applied to GSH determination in *C. elegans*.

#### 4.1.3. Evaluation of Biological Molecules Damage

Overproduction of ROS can lead to oxidative damage of molecules like proteins, lipids or DNA, which may be concomitant and result in cell disorders and ageing. Among them, proteins are possibly the most immediate way for causing cell oxidative damage, owing to their usual role as catalysts [80]. In determining whether to use lipids, DNA or proteins as oxidative stress markers, the nature of the ROS is important. For example, HOCl induces protein carbonylation, but it hardly affects DNA or lipids, which are in turn more sensitive to other ROS. Therefore, when HOCl is the predominant ROS, proteins should be preferred as markers [80].

##### Protein Oxidation

All types of ROS, either radical or nonradical species can oxidize proteins, leading to protein carbonylation (aldehydes and ketones) on specific amino acid side chains, such as lysine, proline, arginine and threonine. These adducts are relatively stable and there exist sensitive methods for their detection, so that their measurement is a common indicator for protein oxidation [80].

The reaction with 2,4-dinitrophenylhydrazine (DNPH) is the most usual approach for the analysis of protein carbonyls and the assessment of protein oxidation in *C. elegans* [81]. This reaction, originally described by Levine et al. [82], results in the formation of a stable 2,4-dinitrophenyl (DNP) hydrazone that can be measured spectrophotometrically, directly or after HPLC separation, or by immunoblotting. In this latter case, quite usually through the commercial OxyBlot detection kit, based on the separation of the DNP-derivatized proteins by SDS-PAGE followed by western blot immunoassay, although it only provides a semiquantitative assessment [80]. DNPH-based assays, including OxyBlot, have been applied to determine protein oxidative damage in *C. elegans* [54,83,84,85,86], although they suffer from poor homogeneity, especially when dealing with complex samples, as it is the case of the worm. An alternative CyDye™-hydrazide-based procedure to quantify protein carbonylation in *C. elegans* has been more recently proposed, based on normalizing carbonyl-related signal to total protein in SDS-PAGE multiplexing experiments and fluorescence scanning in a Typhoon biomolecular imager [87]; according to the authors, this approach improves the performance of classical OxyBlot.

Fluorescein-5-thiosemicarbazide (FTC) is a fluorescent probe that has been used for the analysis of carbonylated proteins in biological samples. It was employed by Chaudhuri et al. [88] for their determination in mouse hepatic tissue after separation of the FTC-protein adducts by 2-D gel electrophoresis with further fluorescence measurement. The same probe was used by Mohanty et al. [89] to analyze carbonylated proteins in plasma using a semi-microplate assay, an approach that was further adapted by Ayuda-Duran et al. [21] to the determination of carbonylated proteins in *C. elegans*.

OxICAT is another technique to evaluate protein oxidation based on the quantification of reversible oxidative thiol groups of proteins, which are a major target of ROS [64]. The method uses an isotope coded affinity tag (ICAT), containing iodacetamide as a trapping reagent; cysteine ICAT-labeled peptides are further analyzed by mass spectrometry [90]. This method was applied to determine the redox status of protein thiol groups throughout *C. elegans* lifespan [91] or the degree of cysteines oxidation in worms submitted to oxidative stress [92].

##### Lipid Peroxidation

Polyunsaturated fatty acids (PUFAs) play a crucial role in signaling and membrane integrity and fluidity. It is well known that PUFAs are highly sensitive to oxidation, leading to the formation of peroxyl radicals (ROO^•^) that are finally cleaved to yield smaller carbonyl molecules, such as malondialdehyde (MDA) and 4-hydroxy-2-nonenal (HNE), which can be used as biomarkers of oxidative damage [1]. HNE is highly electrophilic and tends to form adducts with macromolecules, such as many regulatory proteins, altering their function [93]; its accumulation has been related with different human diseases, and especially neurodegenerative diseases [94]. HNE-modified proteins can be measured by competitive enzyme-linked immunosorbent assay (ELISA). This approach was applied to determine HNE levels in *C. elegans*, in relation to oxidative and free radical damage [95], longevity [96,97] or adipogenesis [98].

Lipid peroxidation products, including HNE and MDA, can be also determined by HPLC after derivatization with 2,4-dinitrophenylhydrazine (DNPH) and further MS detection. This method has been used by Sánchez-Blanco et al. [99], in studies on the influence of *C. elegans* diet in longevity, and by Ayuda-Durán et al. [21] for the assessment of the effect of catechins on worm response against oxidative stress.

Another marker used to evaluate lipid oxidation is lipofuscin. This pigment has a heterogenous composition mainly consisting of oxidized proteins, which may be cross-linked by lipid peroxidation products, like HNE, and different lipids components. Lipofuscin aggregates accumulate during the normal ageing process, so that it is also called the ‘age pigment’, being considered the best marker of cellular ageing. It is particularly found in postmitotic cells, such as neurons and cardiac myocytes [100]. The most usual methods for lipofuscin detection are based on the observation of its autofluorescence by fluorescence microscopy. This has been used to measure lipofuscin accumulation in *C. elegans* in the study of the ageing process [95,101], cellular senescence and longevity [102], or the effects of polyphenols on oxidative stress [60,103].

Isoprostanes are prostaglandin-like end products from non-enzymatic peroxidation of PUFAs that are used as markers of lipid peroxidation in mammals. Different PUFAs give rise to the formation of different isoprostanes. In *C. elegans*, the predominant PUFA is eicosapentaenoic acid, leading to the formation of F3-isoprostanes. An LC-MS method for the analysis of F3-isoprostanes using triple-quadrupole MS detection in multiple reaction monitoring (MRM) mode was developed by Labuschagne et al. [104] and applied to the quantification of endogenous oxidative damage in *C. elegans*. This method measures F3-isoprostanes in whole worms and, therefore, does not contribute information on the localization of the damage. The authors overcame this limitation by using different mutant strains lacking ROS scavenging enzymes expressed in specific tissues or particular cell compartments, so that information on the localization could be deduced. An advantage of using isoprostanes as markers of oxidative damage is that they are chemically stable, contrary to other products from lipid peroxidation like MDA or HNE [64].

##### DNA Damage

8-Hydroxy-2′-deoxyguanosine (8-OHdG or 8-oxodG) is the most common marker for measuring the oxidative damage on DNA, although it has not been much used in *C. elegans*. Isolation of DNA from *C. elegans* homogenates, followed by hydrolysis and dephosphorylation, and further analysis of 8-OHdG by HPLC with electrochemical detection was employed by Arczewska et al. [105] to study the genomic stability and DNA mechanisms of repair in *C. elegans*. An LC-MS/MS method proposed by Yue et al. [106] for the analysis of 8-OHdG in rat liver was adapted by Delgado [107] to evaluate the ability of flavonoids to modulate the oxidative stress response in *C. elegans*, using MRM detection, monitoring the transition from the signal at *m/z* 284 ([M + H]^+^ pseudomolecular ion of 8-OHdG) to the fragment ions at *m/z* 168 and 140. The level of 8-OHdG can also be determined by ELISA using the commercial kit EpiQuik™ (Epigentek, Farmingdale, NY), an approach also employed by Delgado [107]. DNA extracted from *C. elegans* lysates was added to the wells of a treated multi-well plate, the 8-OHdG was captured by adding specific antibodies and the signal enhanced to be detected spectrophotometrically at 450 nm in a microplate reader. It is to say that in the indicated studies, hardly significant changes in the 8-OHdG levels were found, questioning the sensitivity of this marker to measure oxidative damage in *C. elegans*.

DNA damage can also be determined by quantitative PCR. The principle is that DNA lesions hamper the progression of the DNA polymerase, which results in decreased DNA amplification, so that the amount of PCR product, compared to that from equal amounts of untreated DNA, is inversely proportional to the extent of DNA damage. The method was adapted to be applied to *C. elegans* using a number of individuals per assay as small as six [108]. Since the primers used are species-specific, the presence of DNA from other genomes (e.g., DNA from *E. coli* OP50 usually employed to feed worms) does not affect the measurement [108]. This method was, for instance, applied to detect DNA lesions and repair capacity in *C. elegans* during ageing [109].

### 4.2. Activity of Antioxidant Enzymes

Oxidative stress can be indirectly modulated by antioxidant enzymes, such as superoxide dismutases (SOD), catalase (CAT), glutathione peroxidases (GPX), thioredoxins (TRX), glutaredoxins (GLRX) or peroxiredoxins (PRDX). SOD enzymes constitute a first defense line removing superoxide, the primary ROS produced by the mitochondrial electron transfer chain, by dismutation into molecular oxygen and hydrogen peroxide. This latter can be then detoxified to water by PRDX, CAT and GPX, expressed in different cell compartments, and peroxidase activity can be further restored through reduction by either TRX or GLRX [110]. This antioxidant system is highly conserved from microorganisms to humans, being activated in response to different stimuli, including oxidative stress.

Enzymes activity can be measured directly by different in vitro assays. SOD activity has been determined based on the inhibition of superoxide-induced lucigenin chemiluminescence [111], CAT activity by the rate of disappearance of hydrogen peroxide monitoring the absorbance decrease at 240 nm [112], and GPX by the oxidation of GSH coupled to the disappearance of NADPH by glutathione reductase monitored at 340 nm [113].

Another approach to determine enzymes activity is the measurement of the enzyme transcription levels by q-PCR or the use of transgenic *C. elegans* expressing the green fluorescent protein (GFP) under the control of the promoter of their marker genes. These methodologies have been employed by different authors to evaluate the influence of polyphenols on *C. elegans* response against thermal and oxidative stress [59,60,61,62,114,115,116]. In general, exposure to these compounds was related with increased activity and upregulation in the expression of antioxidant enzymes.

Finally, oxidative stress can be also evaluated through the activity of aconitase, an enzyme particularly sensitive to oxidative damage. Aconitase activity can be measured from the conversion of citrate into α-oxoglutarate coupled with the reduction of NADP, followed over time at a wavelength of 340 nm [117]. This method has been applied to measure oxidative stress in *C. elegans* [116], although it has been indicated to lack sensitivity [64].

### 4.3. Exploring Genes and Signaling Pathways Involved in Antioxidant Response 

#### 4.3.1. Mutant Worms

The use of worms with loss-of-function mutations in genes involved in stress or ageing pathways is an interesting approach to explore the antioxidant effects of phytochemicals. A range of well-established gene mutations leading to phenotypes useful to evaluate the oxidative damage have been long described. For example, mutations in *ctl-1* (codifying a cytosolic catalase) are known to enhance oxidative damage, and *ctl-1* knockout mutants accumulate lipofuscin granules causing early ageing [33]; mutants with a deletion in *sod-1* gene, which encodes Cu/Zn SOD in *C. elegans*, show an increase in cytosolic and mitochondrial O_2_^•^^−^ levels [118]; strains carrying a null allele of the thioredoxin *trx-1* gene have shorter lifespan than the wild-type strain [119], and *mev-1* mutants, with a mutation that affects the cytochrome b of the mitochondrial succinate dehydrogenase, accumulate high ROS levels and have decreased lifespan [120]. This latter strain was, for instance, used by Pietsch et al. [121] to assess the effect of caffeic acid, finding that it increased mean lifespan of the mutant, thus suggesting its ability to counteract oxidative stress. Similar observations were made in the same strain when treated with purple *pitanga* (*Eugenia uniflora L*.) extracts, epi- gallocatechin-3,*O*-gallate (EGCG) or myricetin, but not with catechin, kaempferol and naringenin [52,85,122,123].

Mutant knockout strains in genes involved in molecular signaling pathways related to stress, such as insulin/IGF-1, c-Jun N-terminal kinases (JNK), PMK-1/p38 MAPK, HSF-1, SKN-1/Nrf2, SIR-2.1, and AAK-2/AMPK pathways, can also be employed to explore the effect of phytochemicals on the response to oxidative stress [124,125,126]. The activation of these pathways promotes, among others, the expression of catalase (*ctl-1*), superoxide dismutase-3 (*sod-3*), metallothionein (*mtl-1*), heat shock proteins (*hsp*), glutathione *S*-transferase (*gst-4*), glutathione peroxidase (*gpx*), γ-glutamylcysteine synthetase (*gcs-1*), glutathione synthetase (*gss-1*), glutathione reductase (*gsr-1*), NAD(P)H:quinone oxidoreductase (NQO-1), or bacterial pathogen defense genes (*lys-7, spp-1*). All these types of mutants have been used by different authors to evaluate the antioxidant effects and molecular mechanisms of action of multiple phytochemicals, including polyphenols [43,44,73,127,128,129].

##### Insulin/IGF-1 Signaling (IIS) Pathway 

The IIS pathway presents many evolutionarily conserved components that regulate ageing and metabolism in different species, including *C. elegans* [18,130], making it a good target to study the effects and mechanisms of action of phytochemicals in the response to different types of stress, such as thermal, oxidative or osmotic stress, hypoxia, heavy metal toxicity or food shortage.

Two key components in the IIS pathway are the *daf-2* and *age-1* gerontogenes, orthologs of the insulin/IGF-1 receptor and phosphatidylinositol kinase-3-OH (PI3K), respectively. The finding that deletion of those genes doubled the half-life of the wild strain was one of the pioneering and most important discoveries in ageing genetics in *C. elegans* [131,132]. Several polyphenols (e.g., quercetin, epicatechin, catechin, icariin, or icariside II) and cocoa extracts were found not to prolong further the lifespan or increase the resistance to thermal stress in different *daf-2* long-lived mutant strains (i.e., *daf-2(e1368), daf-2(e1370),* or *daf-2(m577)*) [21,22,24,122,133,134]. By contrast, curcumin [135], caffeic acid [121] or flavonoid extracts from *Radix tetrastigma* [136] were reported to continue producing an increase in lifespan and/or stress resistance in *daf-2* mutants. As for the long-lived mutant *age-1(hx546)*, it was found that catechin, caffeic acid or epicatechin-3-gallate were able to increase lifespan or stress resistance [70,121,122], while quercetin, epicatechin, curcumin or chlorogenic acid did not [21,22,24,135,137]. It seems pertinent to point out that the observation of a change in the response of a mutant as a result of the exposure to the phytochemical (e.g., an increase in the resistance against oxidative stress) suggests that the effects induced by that compound are independent of the explored gene. On the contrary, no changes in the mutant behavior would indicate that the gene is required for the effect of the phytochemical.

As previously discussed, DAF-2/IGFR and AGE-1/PI3K activation results in the upregulation of the serine/threonine kinases PDK-1, AKT-1, AKT-2 and SGK-1. The ability of different polyphenols to modulate these transcription factors has been studied using mutant strains. For instance, catechin did not increase the lifespan in the *akt-2(ok393*) mutant [122], and neither did it chlorogenic acid in *pdk-1(sa680), akt-1(ok525), akt-2(ok393)* and *sgk1(ok538)* mutants [137]. Curcumin was not found to enhance oxidative stress resistance in *akt-1(mg144)* and *pdk-1(mg142)* mutants [135], nor epicatechin and quercetin in *akt-1(mg306*) and the double mutant *akt-2(tm812);sgk-1(ft15)* [21,22]. Pietsch et al. [24], however, reported that quercetin was able to prolong lifespan in the *akt-2(ok393)* mutant. A possible explanation for this divergent result could be that *akt-2* mutants are less stress-resistant than *sgk-1* mutants, and this latter gene could be required for the lifespan-extending effects of quercetin.

DAF-16, the ortholog of mammalian FoxO transcription factor, plays a crucial role as a key regulator in the downstream insulin signaling pathways, including stress response and ageing [42,138]. Multiple works aiming to understand the mechanisms behind the polyphenols effects have been performed using mutant strains lacking *daf-16* gene or DAF-16 target genes, such as *sod-3, ctl-1, ctl-2* [42,139] or *dod-3* [140]. Contradictory observations have been made regarding the involvement of DAF-16 in the effects of polyphenols. In studies with different *daf-16* loss-of-function mutant strains, some authors observed that the increase in lifespan and stress resistance induced by phenolic compounds like quercetin [22,24] or catechin [122] was independent of *daf-16*, while others concluded that this transcription factor was required to explain such effects, as in the cases of epicatechin [21], EGCG [70] or several polyphenol-rich plant extracts [85,136,141].

Some authors have also explored the possible implication of JNK-1 in the polyphenols’ effects, as this transcription factor may also activate DAF-16 in parallel with the IIS pathway [124]. Studies with the *jnk-1(gk7)* mutant showed that the life prolonging effects of catechin [122] and quercetin [24], or the improvement in the resistance against oxidative stress of extracts from *Açai* (*Euterpe oleracea*) [142] or *carqueja* (*Baccharis trimera*) [143] were independent of JNK-1.

##### Nrf2/SKN-1 Signaling Pathway

The *skn-1* gene is involved in *C. elegans* in the regulation of a range of detoxification and cell protection processes [49]. Loss-of-function *skn-1* mutants are sensitive to oxidative stress and have a shorter lifespan, while overexpression of *skn-1* contributes to increase longevity and resistance to stress [44,128,138].

Studies with the *skn-1(zu67)* mutant have concluded that Nrf2/SKN-1 signaling could be involved in the protective effects against oxidative stress or lifespan extension induced by epicatechin [21], curcumin [135] or *Hibiscus sabdariffa* L. extracts [141]. However, *skn-1* appears not to be involved in the same effects in the cases of myricetin [25], catechin [122] or *Açai*, *carqueja* and blueberry polyphenols [143,144,145]. Regarding quercetin, Ayuda-Durán et al. [22] observed that the treatment with this flavonol did not improve the survival of the *skn-1(zu67)* mutant when exposed to thermal stress, suggesting that *skn-1* was a mediator in the protective effects of quercetin against stress. However, Pietsch et al. [24] found that quercetin induced an increase in lifespan of the same mutant under normal growth conditions, indicating that, in the absence of stress, this effect was independent of *skn-1*. It has been reported that SKN-1 extends lifespan independently of DAF-16, while it regulates resistance to oxidative stress and detoxification gene expression in response to a reduced IIS signal [44,146]. This dual function of *skn-1* could explain the different results obtained for quercetin by Ayuda-Durán et al. [22] and Pietsch et al. [24].

When IIS is reduced, nuclear accumulation of SKN-1 is dependent on p38 MAPK signaling through NSY-1/SEK-1/PMK-1 pathways [44]. For this reason, loss-of-function mutants of MAPK pathways have also been used to study the involvement of *skn-1* in the effect of polyphenols. Bonomo et al. [144] found that the survival of *nsy-1(ag3)* mutant increased after treatment with *Açai* extracts, but not that of the mutant *sek-1(km4)*, indicating that the antioxidant effect of this fruit might act via the p38 pathway, although only through SEK-1. Pietsch et al. [24] also suggested that SEK-1 could be required for the increase of lifespan mediated by quercetin, whereas Guha et al. [147] did not find that it was needed to explain cranberry-induced longevity.

SEK-1 can be coupled with OSR-1, through UNC-43/CaMKII, to promote resistance to osmotic stress. Thus, many authors have also explored these pathways together. OSR-1/UNC-43/SEK-1 pathway has been proposed as a target for blueberry polyphenols, as they were not able to prolong lifespan in *osr-1(rm1), sek-1(ag1)* and *unc-43(n1186)* mutants [145]. Similarly, *Açai* extracts failed to increase oxidative stress resistance in *osr-1(rm1)* and *unc-43(n498n1186)* mutants [144], and the longevity and thermotolerance enhancing properties of caffeic and rosmarinic acids [121] and curcumin [135] were found to be mediated through regulation of *osr-1, sek-1* and *unc-43*. By contrast, those genes were not relevant for the lifespan extending effects of catechin [122], and *unc-43* but not *osr-1* was required to explain the life extension produced by quercetin [24]. The improvement in oxidative stress resistance induced by *carqueja* polyphenols was also reported to be independent of the MAPK stress-related signaling pathways [143].

##### Heat Shock Protein Response 

Thermal and other environmental stresses activate small heat shock proteins (sHSP), which avoid protein aggregation acting as molecular chaperones and proteases. The *hsp* genes are mainly regulated by heat shock transcription factor 1 (HSF-1), although it could not be a key factor for all sHSP [148].

Many studies have been performed to explore polyphenols mechanisms of action using *hsf-1* mutants, while studies on *hsp* mutants are less numerous. The lifespan extending effects of icariside II and acacetin-rhamnoxyloside were lost in the *hsf-1(sy441)* mutant, suggesting that they were HSF-1 dependent [133,149]. In studies on the same mutant, it was also found that the enhanced stress resistance induced by epicatechin [21] or chlorogenic acid [137] could be mediated by HSF-1. By contrast, quercetin continued producing an increase in the resistance to thermal stress in the *hsf-1(sy441)* mutant, indicating that this effect was independent of HSF-1 [22]. These latter authors also found that treatment with quercetin did not improve the survival in *hsp-16(gk249)* mutants exposed to thermal stress, suggesting that *hsp-16.2* would be necessary to explain the effect of this flavonol in worm resistance against stress [22]. Zhang et al. [150], using the same mutant strain also showed that upregulation of HSP-16.2 was involved in the enhanced heat stress resistance produced by quinic acid, although this factor was dispensable to extend worm lifespan under normal growth conditions.

#### 4.3.2. Transgenic Worms Containing Reporter Gene Fusions

The characteristics of transparency and thinness made *C. elegans* a perfect model to study protein expression in vivo through the use of different reporters, such as green fluorescent protein (GFP), βGAL (LacZ), *Discosoma* sp. red fluorescent protein (dsRED) or yellow fluorescent protein (YFP), being GFP the most usually employed one. Reporters can provide an accurate representation of a gene’s expression pattern. In addition, they can also contribute information about subcellular location and temporal aspects of gene regulation.

There are two main types of reporter gene constructs, transcriptional reporters and translational reporters, which give different information about the expression of a gene, so that choosing the adequate type of reporter is a crucial aspect in gene expression studies. The transcriptional reporters consist of a promoter fragment from a gene of interest driving GFP that provide a tentative expression pattern of the gene under study [39]. The constructs *Pgst-4::gfp*, *Phsp-16.2::gfp* or *Psod-3:gfp* are examples of this type of reporters that have been employed to explore the molecular mechanisms of action of polyphenols in *C. elegans* [21,22,136,143]. The translational reporters are in-frame gene fusions between GFP and a gene of interest. Ideally, a translational reporter includes the entire genomic locus of a gene and preferably should not be disrupting the protein function or topology [39]. DAF-16::GFP and SKN-1::GFP are two of the most used reporters in the study of phytochemicals activity in *C. elegans* [21,22,85,151].

The main transcription factor of IIS pathway, DAF-16, has been widely studied using transgenic strains, in particular the translational reporter *Pdaf-16::daf-16::gfp*, which allows to visualize the subcellular location of DAF-16 using a fusion protein under the control of the *daf-16* promoter. There are numerous references where DAF-16::GFP is analyzed in *C. elegans* after treatment with several polyphenols, reporting different results. In some cases, an increase in the nuclear accumulation of DAF-16 was found [52,123,142], while in others no changes in the subcellular location were observed [21,22,144,152]. Many authors have also studied the promoter activity of the well-characterized DAF-16 target gene *sod-3*, using a reporter strain expressing GFP under the control of the *sod-3* promoter. With this approach, the ability of diverse flavonoids, such as myricetin, quercetin, kaempferol, naringenin or EGCG, to upregulate *sod-3* expression was shown [52,123,150].

The *Pskn-1::skn-1b/c::gfp* transgenic strain has been used to observe intestinal nuclear location of the fusion protein SKN-1::GFP. Thereby, when GFP fluorescence is detected in the intestinal nuclei the signaling pathway is classified as active. It has been found, for instance, that the treatment with myricetin [25] or leaf extract of *Caesalpinia mimosoides* [151] did not change the location of the SKN-1. However, the exposure to *Glochidion zeylanicum* leaf extract induced a significant translocation of SKN-1::GFP [153]. GST-4, an isoform of glutathione S-transferases regulated by the SKN-1 transcription factor was also explored. Using the transgenic strain *Pgst-4::gfp*, it was demonstrated that the treatment of worms with curcumin [135], epicatechin [21] or *Glochidion zeylanicum* leaf extract [153] increased GST-4 expression. However, no change in GST-4 expression was found for quercetin [22] or leaf extract of *Caesalpinia mimosoides* [151].

The gene *gcs-1*, which encodes the enzyme that synthetizes GSH, was also studied using a transgenic strain (*Pgcs-1::gfp*). Paiva et al. [143] observed that extracts from *Baccharis trimera* reduced *Pgcs-1::gfp* expression under stress conditions, but not under normal growth conditions. Similarly, extracts of the *Açai* fruit prevented the upregulation of *Pgcs-1::gfp* under oxidative stress conditions [144]. As above discussed, PMK-1/p38 MAPK directly participates in oxidative stress responses through phosphorylation of SKN-1/Nrf2. Using the transgenic strain [pmk-1(km25) IV; acEx102], Li et al. [136] observed that the treatment with flavonoids from *Radix Tetrastigma* reversed the suppression of the intestinal expression of PMK-1::GFP produced by paraquat.

Transgenic strains (e.g., *Phsp-16.2::gfp, Phsp70::gfp; Phsp60::gfp; Phsp6::gfp*, or *Phsp4::gfp*) have also been used to monitor the expression of different small heat shock proteins, constituting an important tool for measuring stress response in living animals. Worms bearing the GFP reporter gene do not express easily detectable GFP under standard conditions. However, in some of these strains, *hsp* are expressed following either heat shock or oxidative stress. Thus, Ayuda-Durán et al. [21,22] studied the influence of quercetin and epicatechin in the expression of *hsp-70* and *hsp-16.2*, using the *Phsp-70::gfp* and *Phsp-16.2::gfp* reporters, for which worms were previously subjected to a heat shock (35 °C, 1 h) and further allowed to recover at 20 °C for 2 h (*hsp-16.2*) or 3 h (*hsp-70*). The effects of flavonoids and other phytochemicals on different *shsp* strains are diverse. Epicatechin was found to enhance the expression of HSP-16.2 and HSP-70 [21], while quercetin increased that of HSP-16.2 in aged worms but not in young worms [22]. For their part, Abbas and Wink [154] found that overexpression of *Phsp-16.2::gfp* induced by the prooxidant juglone was inhibited by EGCG treatment.

#### 4.3.3. RT-qPCR

One of the most useful and powerful methods to quantify gene expression is the reverse transcription combined with the polymerase chain reaction (RT-PCR). This technology has been adapted to quantitative purposes (RT-qPCR), calculating the relative-fold changes through the 2^−ΔΔCt^ method [155].

There are multiple studies using RT-qPCR to quantify the expression in *C. elegans* of important components from different key pathways as influenced by the treatment with different phytochemicals. Using this technique, the upregulation of *daf-16* and *skn-1* expression by EGCG was shown by Zhang et al. [56]. Also, Ayuda-Durán et al. [21] found that epicatechin enhanced *daf-16, hsf-1* and *skn-1* mRNA levels. Genes related to heat shock proteins have also been a target in these studies. Thus, Pietsch et al. [121] explored the influence of quercetin, caffeic acid and rosmarinic acid in the expression of *hsp-3, hsp-12.6, hsp-16.1, hsp-16.41, hsp-17, hsp-70*, finding that rosmarinic acid upregulated all the six studied *hsp* genes, by four in the case of quercetin, whereas the treatment with caffeic acid resulted in upregulation of only one gene (*hsp-12.6*) and downregulation of the five remaining ones.

The analysis of the expression of multiple genes either in knockout mutants or wild-type worms can be used for categorizing pathways and deciding on their involvement in the effects of phytochemicals. For instance, Bonomo et al. [144] compared the expression of target genes of DAF-16 in *daf-16* knockout mutants and wild-type worms treated with *Açai* extracts, observing that the transcripts levels of *ctl-1* and *gst-7* were upregulated in wild-type animals, but not in the *daf-16* mutant, which allowed them to conclude that upregulation of *ctl-1* and *gst-7* and, therefore, the increase in stress resistance provided by *Açai* was dependent upon *daf-16*. Another possibility is the study of multiple genes regulated by different pathways. Thus, Zheng et al. [137] measured the influence of chlorogenic acid in the expression of DAF-16-targeted genes (i.e., *sod-3, dod-3, hsp-12.6, hsp-16.1* and *hsp-16.2*) and also the mRNA levels of genes regulated by HIF-1 and SKN-1, such as *skn-1, hif-1, gst-4, f22b5.4*, and *nhr-57*.

RT-qPCR was also used to quantify gene expression in *C. elegans* treated with distinct polyphenols and submitted to different stress conditions. Ayuda-Durán et al. [21] found that *daf-16* and *hsf-1* mRNA levels were enhanced in worms grown in the presence of epicatechin, either subjected or not to thermal stress, while *skn-1* was only overexpressed in epicatechin-treated worms under stress, but not in normal growth conditions. On the other hand, Abbas and Wink [156] showed that the expression of *hsp-16.1* and *hsp-16.2* in worms submitted to juglone-induced oxidative stress was lower when they were cultured in the presence than in the absence of EGCG.

## 5. Final Remarks

Many in vitro assays have been developed to measure the antioxidant activity of phytochemicals, based on different mechanisms of reaction. However, such assays do not adequately reflect the in vivo situation, so that their results have to be taken with caution and conjugated with other aspects, such as compounds bioavailability and tissue and cell environment.

The nematode *Caenorhabditis elegans* is a well-characterized and easy to manipulate model organism that offers a suitable alternative for the in vivo study of the effects and mechanisms of action of plant secondary metabolites. As above discussed, stress-related biological targets, organism responses and molecular pathways prevailing in mammals can be explored using this worm. It has been estimated that 60–80 % of *C. elegans* genes possess human homologous, and many biological processes, including response against oxidative stress, ageing, apoptosis, cell signaling, metabolism, or cell cycle are conserved between the worm and humans [37]. An outstanding feature of *C. elegans* is its genetic flexibility. Many mutant (knock out) strains are available, and new ones can be easily generated through knocking down (RNAi) technology. This opens the possibility to produce tailored models for specific purposes, such as inducing a disease-related phenotype, reproducing molecular disease mechanisms or performing molecular mechanistic studies with dietary interventions. Indeed, *C. elegans* fills in the gap between in vitro and in vivo approaches, allowing a high-throughput reductionist approach, providing at the same time physiologically relevant data derived from a whole-animal [37,157].

A clear advantage of *C. elegans* over other animal models derives from its regulatory status. Despite it is not true, it is not legally defined as an animal in European, USA or Canadian conventions, which excludes it from ethical limitations for its experimental usage, contrary to vertebrates, like mice, rats or zebrafish, that are protected by stringent animal rights regulations [157]. A summary of the main characteristics of *C. elegans* compared with other ex vivo and in vivo models is collected in the Table 2.

Despite its many advantages, *C. elegans* is not free from limitations. As a nematode, it is biologically far from mammals and, therefore, its capacity to predict toxicity or efficacy in humans is not perfect. Furthermore, some molecular pathways do not exist in the worm, and therefore cannot be studied there. Thus, it should be rather seen as an amenable model for the elucidation of mechanisms of action and/or a fast screening system to be used in early research to deliver quick answers to specific problems, like establishing the function of a gene, or getting preliminary information that allow more documented and cost-effective late preclinical developments in murine models [37]. Regarding antioxidant assessment, different methodological approaches providing complementary information can be employed to evaluate the role of phytochemicals using the C. elegans model. The most direct one is the observation of the phenotypical modifications after submitting the worms treated with a given compound or extract to an oxidative challenge. Further information on the molecular targets can be obtained from the analysis of chemical and biochemical markers, such as ROS, carbonylated proteins, lipid peroxides, altered DNA or the activity of enzymes related with the endogenous defenses against stress. In addition, *C. elegans* also offers the possibility to explore genes and signaling pathways involved in the regulation of the oxidative stress through different strategies, like the use of worms carrying loss-of-function mutations or transgenic strains containing reporter gene fusions, as well as the analysis of gene expression by RT-qPCR. These methodological approaches are summarized in Table 3.

Using those methodologies, the antioxidant and stress-modulating abilities of diverse phytochemicals, and particularly polyphenols, have been explored. As a general conclusion, the reported results revealed that there is not a common mechanism of action that can be ascribed to all polyphenols and circumstances. Whereas some of the different (and sometimes contradictory) results obtained by different authors might be explained by the distinct approaches and assay conditions employed, more studies are required to produce a clear idea of the mechanisms and molecular pathways involved in the antioxidant effects and biological activity of polyphenols in living organisms. In the coming years, novel methodological developments and further advances in the knowledge in this field can be expected, for which *C. elegans* should continue showing as a convenient and invaluable model.

## Figures and Tables

**Table 1:** ROS determination probes.

Probe	Reaction	Specificity	Limitations
DCFH-DA	The colourless reduced form DFCH is oxidized to fluorescent DCF	Sensitive to H_2_O_2_, ^•^HO and ROO^•^	No detection of ^•^NO, HOCl or O_2_^•^^−^
MitoTracker^®^ red CM-H(2)XRos	Oxidation of the reduced form to the red-fluorescent dye rosamine	Especially H_2_O_2_	Poor detection of other ROS
MitoSOX™	Dihydroethidium (DHE) is oxidized by O_2_^•^^−^ to the fluorescent ethidium form	Mainly O_2_^•^^−^	Possible reaction with cell components like cytochrome C
Amplex red	Formation of fluorescent resorufin upon oxidation of 10-acetyl-3,7-dihydroxy- phenoxazine	H_2_O_2_	Interference of reductants like glutathione or NADH. No detection of intracellular H_2_O_2_

**Table 2:** Main characteristics of different ex vivo and in vivo models (information adapted and extended from Calvo et al. [157]).

	*C. elegans*	Cell Cultures	Yeasts	*Drosophila melanogaster*	Zebra Fish (*Danio rerio*)	Murine Models
Handling and maintenance	Easy	Easy	Easy	Fair	Fair	Difficult
Consideration of bioavailability issues	Yes	No	No	Yes	Yes	Yes
Throughput	High	High	High	Moderate	Good	Low
Availability of disease models	Good	Good	Limited	Good	Limited	High
Human prediction capacity	Moderate	Poor	Poor	Poor	Moderate	Good
Ease for genetic manipulation	Good	Good	Good	Good	Limited	Poor
Ethical concerns	No	May exist	No	Yes	Yes	Yes
Drawbacks	Biologically far from mammals Primitive immune system	Not a physiological setting	Biologically far from mammals Low degree of homology with human genes	Difficult to scale and handling system (it flies)	Difficult testing of non-soluble molecules	Facilities and breeding requirements

**Table 3:** Methodologies that can be used for the evaluation of the antioxidant potential in the *C. elegans* model.

Approach	Procedures	Observations
Phenotypical assessment	Evaluation of the survival or phenotypical modifications in worms treated with the compound after submission to an oxidative challenge (e.g., paraquat, H_2_O_2_, juglone, thermal stress)	Results highly by assay conditions (analyte concentration, treatment conditions, worm age or strength of the oxidative challenge)
Markers of oxidative damage		
1. ROS	Measurement after reaction colored or fluorescent probes: dichlorofluorescein, MitoTracker^®^ red CM-H(2)XRos, MitoSOX™, Amplex red	Different probes have different specificity towards different probes
2. Glutathione	Spectrophotometric or HPLC analysis after reaction with DTNB or OPA)	Determination of total glutathione (i.e., GSH + GSSG) requires previous GSSG reduction by glutathione reductase.
3. Carbonylated proteins	Reaction with 2,4-dinitrophenyl hydrazine (DNPH) or fluorescein- 5-thiosemicarbazide (FTC). Spectrophotometrical HPLC, or immunoblotting (OxyBlot assay) measurement	Poor homogeneity Semiquantitative assessment (OxyBlot)
4. Lipid oxidation products	LC-MS or ELISA analysis of lipid degradation products (MDA, HNE, isoprostanes). Assessment of lipofuscin accumulation by fluorescence microscopy	Different stages of the lipid oxidation are evaluated depending on the approach
5. DNA damage	Measurement of 8-OHdG spectrophotometrically or by LC-MS/MS	Low sensitivity
Antioxidant enzymes	Measurement of the activity of different enzymes (e.g., SOD, CAT, GPXs, TRXs, GLRXs, PRDX, aconitase) typically in a microplate reader	Indirect measurement Different enzymes measure different processes Low sensitivity
Mutant worms	Assessment of the behavioral responses of worms with loss-of-function mutations in genes belonging to conserved stress or ageing pathways (e.g., insulin/IGF-1, SKN-1/Nrf2) treated with the compound.	Suited for evaluation of molecular mechanisms of action Highly variable results depending on the assay conditions.
Transgenic worms carrying fluorescent reporters	Microscopy observation of the fluorescence of different reporters: green fluorescent protein (GFP), βGAL (LacZ), *Discosoma* sp. red fluorescent protein (dsRED), yellow fluorescent protein (YFP)	Allow detection of subcellular location
RT-qPCR	Quantitative measurement of changes in expression of a gene	Information about the expression of a particular gene
